# *Panax notoginseng* saponins (PNS) attenuate Th17 cell differentiation in CIA mice via inhibition of nuclear PKM2-mediated STAT3 phosphorylation

**DOI:** 10.1080/13880209.2023.2173248

**Published:** 2023-02-16

**Authors:** Mei-Yu Shen, Yu-Xi Di, Xiang Wang, Feng-Xiang Tian, Ming-Fei Zhang, Fei-Ya Qian, Bao-Ping Jiang, Xue-Ping Zhou, Ling-Ling Zhou

**Affiliations:** a School of Pharmacy, Jiangsu Key Laboratory for Pharmacology and Safety Evaluation of Chinese Materia Medica, Nanjing University of Chinese Medicine, Nanjing, Jiangsu Province, People's Republic of China; b Department of Rheumatology, Affiliated Hospital of Nanjing University of Chinese Medicine, Nanjing, Jiangsu Province, People's Republic of China; c The First Clinical Medical College, Nanjing University of Chinese Medicine, Nanjing, Jiangsu Province, People's Republic of China

**Keywords:** Immunometabolism, CD4^+^T cells, glycolysis, autoimmune disease, *Panax notoginseng* saponins

## Abstract

**Context:**

Rheumatoid arthritis (RA) is an autoimmune disease with aberrant Th17 cell differentiation. *Panax notoginseng* (Burk.) F. H. Chen (Araliaceae) saponins (PNS) have an anti-inflammatory effect and can suppress Th17 cell differentiation.

**Objective:**

To investigate mechanisms of PNS on Th17 cell differentiation in RA, and the role of pyruvate kinase M2 (PKM2).

**Materials and methods:**

Naive CD4^+^T cells were treated with IL-6, IL-23 and TGF-β to induce Th17 cell differentiation. Apart from the Control group, other cells were treated with PNS (5, 10, 20 μg/mL). After the treatment, Th17 cell differentiation, PKM2 expression, and STAT3 phosphorylation were measured *via* flow cytometry, western blots, or immunofluorescence. PKM2-specific allosteric activator (Tepp-46, 50, 100, 150 μM) and inhibitor (SAICAR, 2, 4, 8 μM) were used to verify the mechanisms. A CIA mouse model was established and divided into control, model, and PNS (100 mg/kg) groups to assess an anti-arthritis effect, Th17 cell differentiation, and PKM2/STAT3 expression.

**Results:**

PKM2 expression, dimerization, and nuclear accumulation were upregulated upon Th17 cell differentiation. PNS inhibited the Th17 cells, RORγt expression, IL-17A levels, PKM2 dimerization, and nuclear accumulation and Y705-STAT3 phosphorylation in Th17 cells. Using Tepp-46 (100 μM) and SAICAR (4 μM), we demonstrated that PNS (10 μg/mL) inhibited STAT3 phosphorylation and Th17 cell differentiation by suppressing nuclear PKM2 accumulation. In CIA mice, PNS attenuated CIA symptoms, reduced the number of splenic Th17 cells and nuclear PKM2/STAT3 signaling.

**Discussion and conclusions:**

PNS inhibited Th17 cell differentiation through the inhibition of nuclear PKM2-mediated STAT3 phosphorylation. PNS may be useful for treating RA.

## Introduction

Rheumatoid arthritis (RA) is an autoimmune disease characterized by synovial inflammation and the formation of pannus, leading to the destruction of joint cartilage and bone (McInnes and Schett 2011; Smolen et al. 2018). The hallmark of inflamed synovium is the infiltration of inflammatory cells into joints, including innate immune cells and adaptive cells (B cells and T cells) (Srivastava et al. 2018). Although the pathogenesis of RA has not been comprehensively defined, it is clear that CD4^+^T helper (Th) cells are implicated in the development of RA, which contain Th1, Th2, Th9, Tfh, Th17, and Treg subsets (Jiang et al. 2020). Importantly, previous studies indicate that the aberrant differentiation of Th17 cells, which contribute to the host defense against extracellular pathogens in normal conditions, has been recognized as a critical component of the initiation and progression of RA (Komatsu et al. 2014; Yang P et al. 2019). In the stimulation of immunoregulatory cytokines such as interleukin 6 (IL-6), interleukin 23 (IL-23), and transforming growth factor-β (TGF-β), naive CD4^+^T cells could differentiate into Th17 cells (Yang et al. 2014). IL-6 drives the phosphorylation of signal transducer and activator of transcription 3 (STAT3), which could induce the expression of Th17 specific transcription factor retinoic acid related orphan receptor γt (RORγt) (Korn et al. 2009). Low concentrations of TGF-β can prolong STAT3 activation and induce RORγt function by assisting IL-6 (Chen et al. 2006). IL-23 finally induces the pathogenicity of Th17 cells by promoting the production granulocyte-macrophage colony-stimulating factor (GM-CSF) (Komuczki et al. 2019). Nevertheless, it remains unclear about the regulatory mechanism that results in the aberrant differentiation of Th17 cells in RA patients.

Recent studies have shown that functionally distinct T cell subsets require distinct metabolic programs to support their specific functional needs (Freitag et al. 2016). Th17 cells were proved to utilize glycolysis to fulfill the metabolic requirements for supporting cellular growth and differentiation (Shi et al. 2011; Buck et al. 2015). Thus, changes in cell metabolism have been shown to enhance or suppress specific T cell functions. Consistent with this, inhibiting glycolysis by targeting glucose-6-phosphate isomerase 1 (Gpi1) could selectively eliminate inflammatory encephalitogenic and colitogenic Th17 cells (Wu et al. 2020).

Pyruvate kinase (PK), a key enzyme of glycolysis, is at the final step of glycolysis and converts phosphoenolpyruvate (PEP) to pyruvate. PK has four isoforms which are distributed in different cell types. Immune cells preferentially express the isoforms PKM1 and PKM2, and PKM2 is apt to express in proliferating cells and is subject to complex allosteric regulation that controls its enzymatic activity (Israelsen and Vander Heiden 2015). In general, PKM2 is present in cells as a tetrameric or dimeric protein. Tetrameric PKM2 locates in the cytoplasm that is highly active enzymatically and efficiently converts PEP to pyruvate. In recent years, non-metabolic functions of dimeric PKM2 have been discovered, such as translocating into the nucleus and regulating gene expression and protein kinase activity (Dong et al. 2016). In particular, dimeric PKM2 was shown to translocate into the nucleus to activate STAT3 in CD4^+^T cells, macrophages, and cancer cells (Ma et al. 2019; Damasceno et al. 2020; Hou et al. 2020). However, the potential mechanisms of PKM2 in T cell activation and differentiation remain unclear. Recent studies reported that inducing PKM2 tetramerization and blocking its nuclear translocation could strongly inhibit CD4^+^T cell activation and pathogenicity (Angiari et al. 2020), and the expression of PKM2 is associated with Th17 cell differentiation (Seki et al. 2020). Therefore, regulating PKM2 conformation to suppress Th17 cell differentiation may be a promising therapeutic strategy for RA.

*Panax notoginseng* saponins (PNS), the key active components of *Panax notoginseng* (Burk) F. H. Chen (Araliaceae), includes more than 30 different types of saponins, among which ginsenosides Rb1, Rg1, and notoginsenoside R1 are found in the highest content (Uzayisenga et al. 2014). It is recognized that these natural products exhibit multiple biological activities, including anti-inflammation, anti-apoptosis, antioxidation, vasodilation, anti-coagulation, etc. (Wang et al. 2016). Although PNS have been found to prevent joint bone destruction in the rabbit antigen-induced arthritis (AIA) model (Wei et al. 2021) and the progression of RA (Li 1991), the potential mechanisms remain unclear. Moreover, recent reports showed that PNS functions as an anti-inflammatory agent by suppressing both proliferation and differentiation of Th17 cells. But whether PNS improves RA *via* inhibiting Th17 cell differentiation and the mechanisms of it are not known.

In this study, we showed that PNS treatment attenuated inflammation and Th17 cell differentiation in mice with the collagen-induced arthritis (CIA) model. Further experiments demonstrated that the dimeric PKM2 translocated into the nucleus and promoted phospho-STAT3 at Y705 throughout the differentiation of Th17 cells. PNS could selectively inhibit dimeric PKM2 and block its nuclear accumulation and severely impacted STAT3 activation and Th17 cell differentiation. Our work suggested that PNS regulating PKM2 conformation can function as a potential therapeutic approach to control Th17-mediated autoimmunity diseases such as RA.

## Materials and methods

### Mice

Male C57BL/6 mice (6–8 weeks old) were purchased from Weitong Lihua Experimental Technology Co., Ltd [Zhejiang, China; Animal license number: SCXK (Zhe) 2019-0001]. Male DBA/1J mice (6–8 weeks old) were obtained from Model Animal Research Center of Nanjing University [Animal license number: SCXK (Su) 2015-0001]. All mice were allowed to acclimate for at least 3 d under specific-pathogen-free conditions with constant temperature (23 °C ± 2 °C), humidity (60% ± 5%) and a 12 h light/dark cycle. Experimental procedures were approved by the Animal Ethics Committee of Nanjing University of Chinese Medicine (No. 202103A010).

### Induction of CIA model and PNS treatment

The CIA mouse model was prepared as previously described (Yang et al. 2020). Male DBA/1J mice used for CIA induction were immunized s.c. at the base of the tail with bovine type II collagen (CII) (Chondrex, 20021) and Freund’s adjuvant (CFA) (Sigma-Aldrich, F5881). The certain amount of CII was mixed with CFA in a 1:1 ratio (v/v) and emulsified at 4 °C. The day of the first immunization was determined on day 0. On day 21, 0.1 mL CII emulsified with incomplete Freund’s adjuvant (IFA) (final concentration of 1 mg/mL) was s.c. in the tail, proximally to the primary injection site, to enhance immunization. For PNS treatment, mice were intragastric administered (i.g.) every day from day 21 to day 49 with 100 mg/kg PNS (Shanghai Yuanye Bio-Technology Co., Ltd., B21102) dissolved in distilled water. The control and model group mice were administered with isopyknic distilled water. The dose of PNS (100 mg/kg) was based on long-term exploration of our lab; arthritis was effectively relieved but no toxic side effects were observed in mice (unpublished). The development of disease was monitored at day 21, 28, 35, 42, 49 by assessing the paw thickness and the arthritis scores. Arthritis scores were scored 0–4. The maximum scores per mice were 16 (4 points × 4 paws). At day 49, all mice were sacrificed by anesthesia, the spleens and serum were collected for further studies.

### Naïve CD4^+^T cell isolation and differentiation

Murine naïve CD4^+^T cells were purified from the spleen of male C57BL/6 mice by magnetic cell negative selection using a mouse naïve CD4^+^T cell isolation kit (Miltenyi Biotec, 130-104-453), according to manufacturer’s instructions. Cells were stimulated *in vitro* with plate-bound anti-CD3 (BD Pharmingen, 553057) and anti-CD28 antibodies (BD Pharmingen, 553294) (both for 2 μg/mL) in RPMI 1640, supplemented with 10% heat-inactivated fetal bovine serum and 100 U/mL penicillin/streptomycin. To induce the differentiation of Th17 cells, cells were activated in the presence of 25 ng/mL IL-6 (Novoprotein, CG39), 20 ng/mL IL-23 (Novoprotein, CI18), 2.5 ng/mL TGF-β (Novoprotein, CA59). PNS, Tepp-46 (Apexbio, C4181), and SAICAR (Apexbio, C3413) were dissolved at a concentration of 10 mM in dimethyl sulfoxide (DMSO) and stored at −20 °C until use. For PNS treatment, IL-6/IL-23/TGF-β were added to each well, followed by treatment with PNS (5, 10, 20 μg/mL) and incubation for 72 h. For Tepp-46 and SAICAR treatment, cells were pre-incubated at 37 °C for 24 h with Tepp-46 (50, 100, 150 μM) or SAICAR (2, 4, 8 μM) before IL-6/IL-23/TGF-β activation. For co-treatment, cells were pretreated with SAICAR (4 μM) or Tepp-46 (100 μM) for 24 h before the condition of Th17-polarization), and treated with PNS (10 μg/mL) for another 72 h.

### Cell counting kit-8 (CCK8) assay

The cells were seeded in 96-well plates at a cell density of 1 × 10^5^/well. Then, 25 ng/mL IL-6, 20 ng/mL IL-23, and 2.5 ng/mL TGF-β were added to each well, followed by treatment with PNS (1.25, 2.5, 5, 10, 20, 40, 80 μg/mL) or Tepp-46 (50, 100, 150, 200, 250 μM) or SAICAR (1, 2, 4, 8, 16, 32 μM). After incubation for 72 h or 24 h, the cell viability was evaluated by CCK8 assays according to the manufacturer’s instructions (Apexbio, K1018).

### Enzyme activity

PK enzyme activity of Th17 cells after PNS (5, 10, 20 μg/mL) treatment was examined using a Pyruvate Kinase Activity Assay kit (Solarbio, BC0540). After treatment for 72 h, all procedures were performed according to the manufacturer’s instructions.

### Quantitative real-time PCR (RT-qPCR)

Total RNA was isolated from CD4^+^T cells using TRIzol^TM^ Reagent (Ambion, 15596026) according to the manufacturer’s instructions and assessed for concentration and purity using absorption ratios of 260/280 nm and 260/230 nm by NanoDrop Microvolume spectrophotometer (Thermo). RNA was converted to cDNA using the reverse transcription kit HiScript Q RT SuperMix (Vazyme, R222-01), and quantification of gene expression was done by real-time PCR performed on resulting cDNA *via* ChamQ Universal SYBR qPCR Master Mix (Vazyme, Q711-02) and an ABI 7500 thermocycler (Applied Biosystems Life Technologies, Foster City, CA, USA). Gene relative expression was calculated by the 2−^ΔΔCt^ and normalized to a reference control (GAPDH). The primers (Sangon Biotech) used in this study were listed in Table 1.

**Table 1.** The primers used for RT-qPCR.

Name	Primers	Sequence 5′–3′
GAPDH	Forward	AGGTCGGTGTGAACGGATTTG
	Reverse	TGTAGACCATGTAGTTGAGGTCA
PKM2	Forward	TGTCTGGAGAAACAGCCAAG
	Reverse	CGAATAGCTGCAAGTGGTAGA
STAT3	Forward	CCGTCTGGAAAACTGGATAACTTC
	Reverse	CCTTGTAGGACACTTTCTGCTGC
RORγt	Forward	ACAAATTGAAGTGATCCCTTGC
	Reverse	GGAGTAGGCCACATTACACTG

### Flow cytometry

All flow cytometry analyses were performed after 72 h post-polarization. For Th17 detection, cells were first re-stimulated with Leukocyte Activation Cocktail (BD Pharmingen, 550583) for intracellular cytokine staining. After 4 h, cells were then washed, fixed/permeabilized with fixation and permeabilization buffers (BD Pharmingen, 554722) and stained with mouse CD4-FITC (145-2C11), mouse IL-17A-PE (TC11-18H10) (all for BD Pharmingen). For analysis and gating, we set up auxiliary staining groups including no staining, single staining, isotype staining. Finally, data were acquired on flow cytometry (Beckman, FC-500) and analyzed by the FlowJo V10 software.

### Molecular docking analysis

Molecular docking was used to investigate the binding mode between PNS and the PKM2 protein with the AutoDock Vina (http://vina.scripps.edu/). The three-dimensional (3D) structures of PKM2 (PDB ID: 1T5A) was downloaded from the RCSB Protein Data Bank (http://www.rcsb.org/pdb/home/home.do). The 3D structures of ginsenoside Rb1, ginsenoside Rg1 and notoginsenoside R1 were downloaded from Traditional Chinese Medicine Database and Analysis Platform (TCMSP, https://tcmsp-e.com/). The default parameters were used as described in the AutoDock Vina. The top-ranked pose as judged by the docking score was subjected to visual analysis using PyMOL 2.2.0 software (http://www.pymol.org/).

### PKM2 crosslinking, nuclear extraction and western blot

For standard western blots, cells were collected and lysed in RIPA buffer with protease and phosphate inhibitors (Beyotime, P1045), followed by mixing with SDS loading buffer and heating at 100 °C for 5 min. For detection of dimeric/tetrameric PKM2 expression, cells were collected, washed twice with phosphate-buffered saline (PBS, pH 8.0) and incubated in 2 mM disuccinimidyl suberate (DSS, Sangon Biotech, C100015) which dissolved in PBS (pH 8.0) for 30 min at 37 °C. Cells were then washed, lysed in RIPA buffer, mixed with SDS loading buffer and heated at 100 °C for 5 min. For nuclear extraction, nuclear and cytoplasmic fractions were isolated from cells by nuclear and cytoplasmic protein extraction kit (Beyotime, P0027), following the manufacturer’s instructions. All the protein content was determined using enhanced BCA protein assay kit (Beyotime, P0010S). The protein samples (10 μg per lane) were loaded on 6% or 10% or 12% SDS-PAGE gels and transferred onto polyvinylidene fluoride (PVDF) membranes. Membranes were blocked with 5% bovine serum albumin (BSA), incubated with PKM2 primary antibody (Proteintech, 15822-1-AP; 1:1000 dilution) or STAT3 primary antibody (CST, D3Z2G; 1:1000 dilution) or phosphor-STAT3 (Tyr705) (CST, D3A7; 1:2000 dilution) overnight at 4 °C and secondary HRP-conjugated antibodies (proteintech, SA00001-2; 1:3000 dilution) for 1.5 h. Images were obtained and analyzed with the Image Lab software (Bio-Rad). Histone H3 and β-actin were used as nuclear and cytoplasm loading controls, respectively.

### Immunofluorescence

For the intracellular detection of PKM2, CD4^+^T cells were washed twice and fixed/permeabilized in permeabilization/fixation buffer (Beyotime, P0096) for 30 min at 4 °C, and blocked with 5% BSA. After washing with PBS, cells were incubated with PKM2 primary antibody (Proteintech, 15822-1-AP; 1:50 dilution) overnight at 4 °C followed by 2 h incubation with 1:200 secondary CoraLite594 conjugated goat anti-rabbit (Proteintech, SA00013-4). One million cells resuspended in 100 μL PBS were counterstained with DAPI to detect nuclei, spun in the cytospin (250 rpm for 5 min) to allow the attachment to the coverslips and then mounted for microscopy. All images were acquired using the thunder imager system (Leica).

### Enzyme-linked immunosorbent assay (ELISA)

The levels of TGF-β, tumor necrosis factor-α (TNF-α, EK282HS), IL-6 (EK206HS), and interleukin 17 A (IL-17A, EK217HS) in mouse serum and cell supernatants were measured by Mouse High Sensitivity ELISA kit (all for MultiSciences) according to the manufacturer’s instructions.

### Statistical analysis

Data were performed as mean ± SEM and statistically significant were considered if *p* < 0.05. Statistical comparisons were determined by one-way ANOVA and two-way ANOVA with Tukey’s *post hoc* test using GraphPad Prism 6 software.

## Results

### PNS reduced Th17 cell differentiation in vitro and in vivo

**Figure 1.**
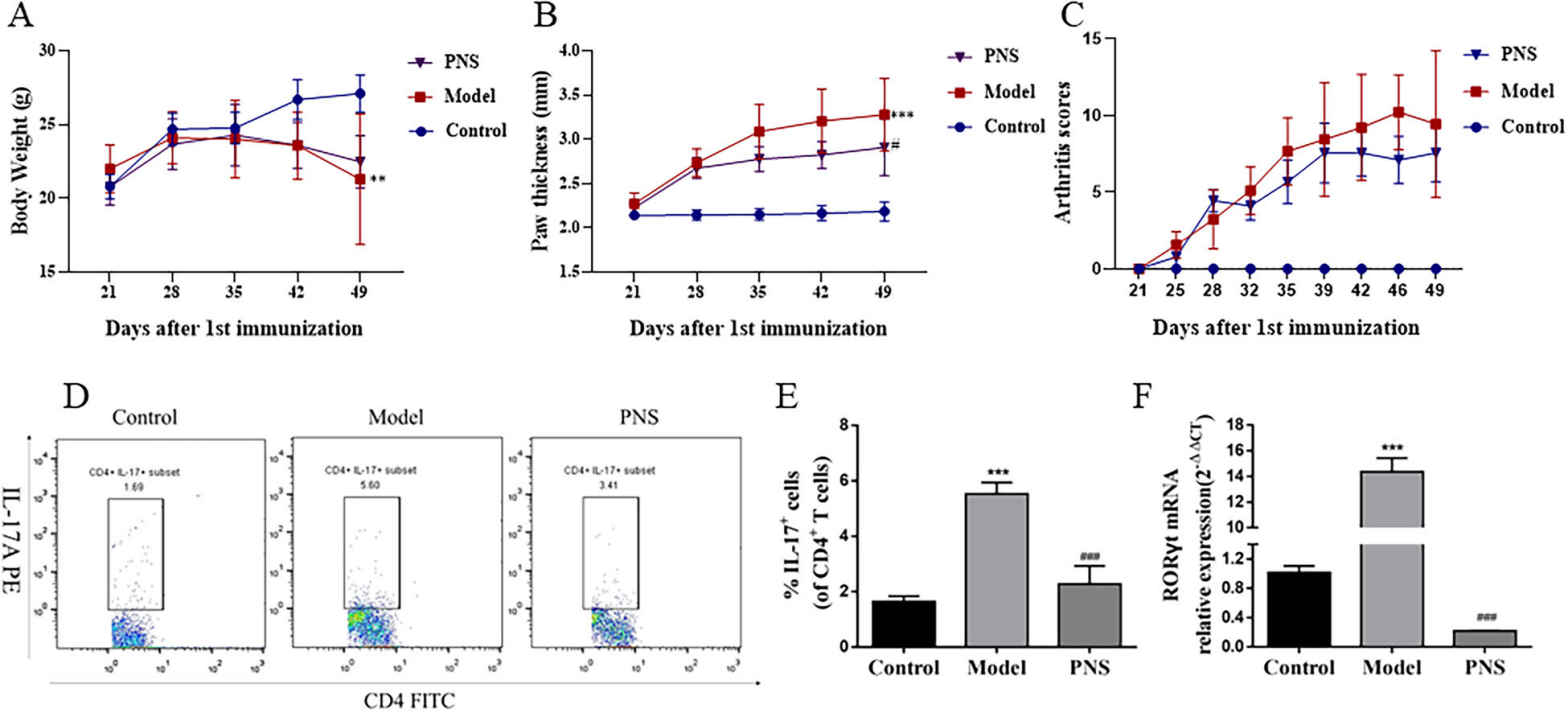
PNS attenuated the clinical severity and Th17 cell differentiation in CIA mice. DBA/1J mice were immunized with bovine type II collagen emulsified in complete Freund adjuvant and incomplete Freund adjuvant. Mice were treated at day 21 after the first immunization with saline or PNS (100 mg/kg). (A–C) Body weight, mean arthritis score and paw thickness in CIA mice treated with PNS. (D,E) The representative dot plots and percentage of CD4^+^IL-17^+^ cells were determined by flow cytometry in the spleen of CIA mice treated with PNS. (F) The mRNA expression of RORγt was evaluated by RT-qPCR in the splenic CD4^+^T cells of CIA mice treated with PNS. Data were expressed as the mean ± SEM (*n* = 6). For (A), (B) and (C), *p < 0.05, **p < 0.01, ***p < 0.001 compared with the control group, ^#^*p* < 0.05, ^##^*p* < 0.01, ^###^*p* < 0.001 compared with the model group, by two-way ANOVA with Dunnett test. For (D) and (F), *p < 0.05, **p < 0.01, ***p < 0.001 compared with the control group, ^#^*p* < 0.05, ^##^*p* < 0.01, ^###^*p* < 0.001 compared with the model group, by one-way ANOVA with Tukey’s *post hoc* test.

The CIA mouse model was successfully established to investigate the effect of PNS on arthritis. The arthritis score and paw swelling in saline-treated model mice increased compared with those of the control, whereas PNS-treated (100 mg/kg) mice had a significant reduction of arthritis score and paw thickness compared with those in model mice (Figure 1(A–C)). To observe the effect on Th17 cell differentiation, the RORγt mRNA expression and the percentage of CD4^+^IL-17^+^ cells in spleen were assessed. Compared with those of the control, the high expression of RORγt and the increase of Th17 cells were observed. We found that PNS (100 mg/kg) treatment markedly inhibited the ratio of Th17 cells and the RORγt mRNA expression (Figure 1(D–F)). These results indicated that PNS attenuated the development of CIA model and Th17 cell differentiation *in vivo*.

**Figure 2.**
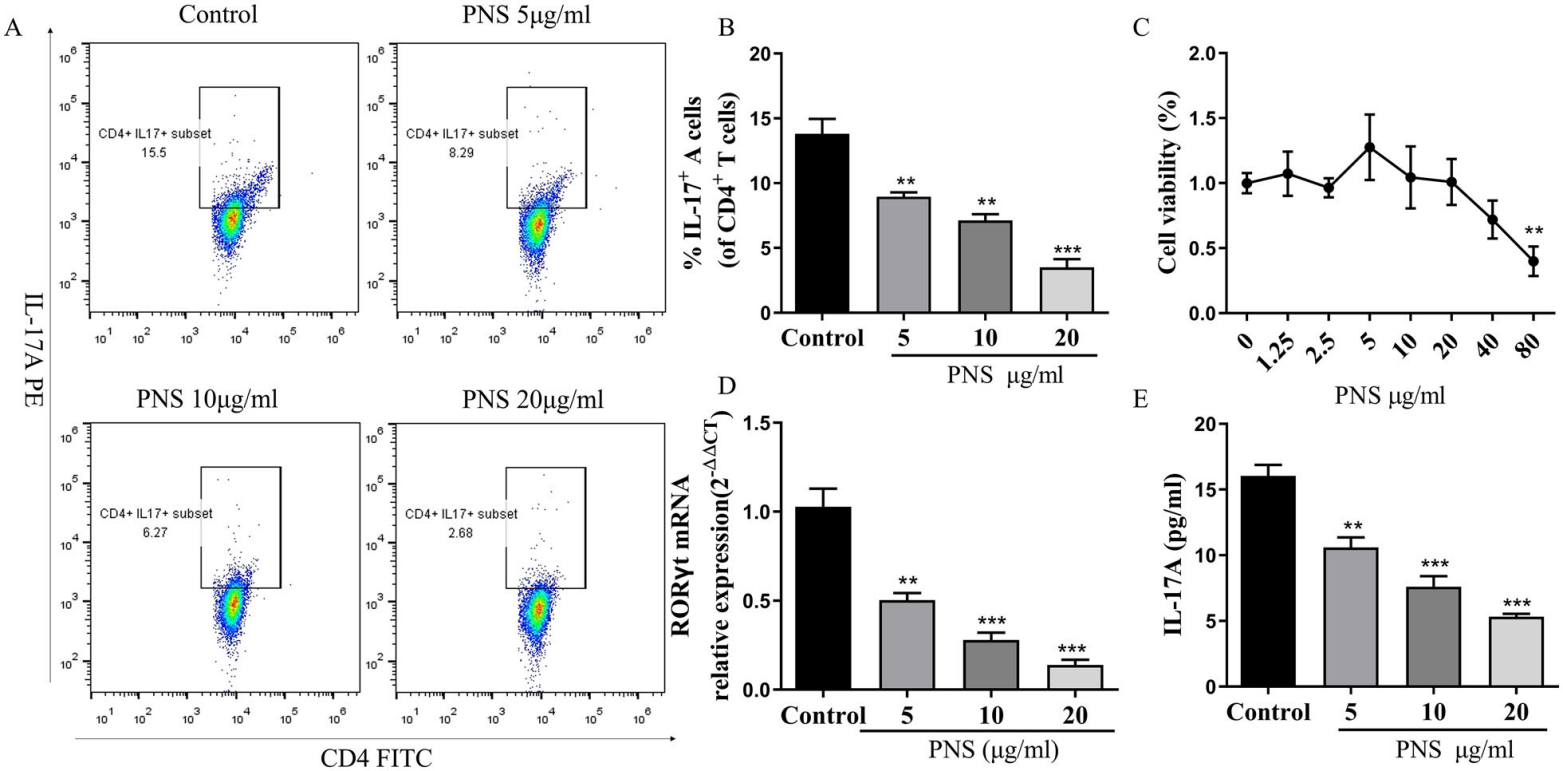
PNS reduced Th17 cell differentiation *in vitro*. (A,B) Representative dot plots and the percentage of Th17 cells (CD4^+^IL-17^+^ cells) (with the condition of Th17-polarization) treated with PNS (5, 10 and 20 μg/mL) were determined by flow cytometry. (C) The cell viability of CD4^+^T cells (with the condition of Th17-polarization) treated with PNS was detected by CCK8. (D) The mRNA expression of RORγt in CD4^+^T cells (with the condition of Th17-polarization) treated with PNS (5, 10 and 20 μg/mL) was evaluated by RT-qPCR. (E) The levels of IL-17A in cell supernatants was determined by ELISA. Data were expressed as the mean ± SEM (*n* = 3). *p < 0.05, **p < 0.01, ***p < 0.001 compared with the control group by one-way ANOVA with Tukey’s *post hoc* test.

To directly investigate the potential effect of PNS on the differentiation of Th17 cells, we cultured the naïve CD4^+^T cells from C57BL/6 mice under induced Th17 cell–polarizing conditions *in vitro*. To test whether PNS exerted cytotoxicity in Th17 cells, we measured cell viability using the CCK8 assay. The results suggested that at ranges of 1.25–20 μg/mL, PNS was not cytotoxic to Th17 cells, whereas 80 μg/mL PNS started to induce cellular toxicity (Figure 2(C)). Then, doses of 5, 10 and 20 μg/mL were selected for subsequent *in vitro* experiments to ensure that the effects of PNS treatment were not due to its cytotoxicity. These data showed that PNS dramatically reduced the percentage of CD4^+^IL-17^+^ cells, the RORγt mRNA expression and the levels of IL-17A (Figure 2(A,B,D,E)), which indicated that PNS were capable to inhibit Th17 cell differentiation *in vitro*.

### PNS targeted PKM2 to inhibit PKM2 dimerization and nuclear accumulation

**Figure 3.**
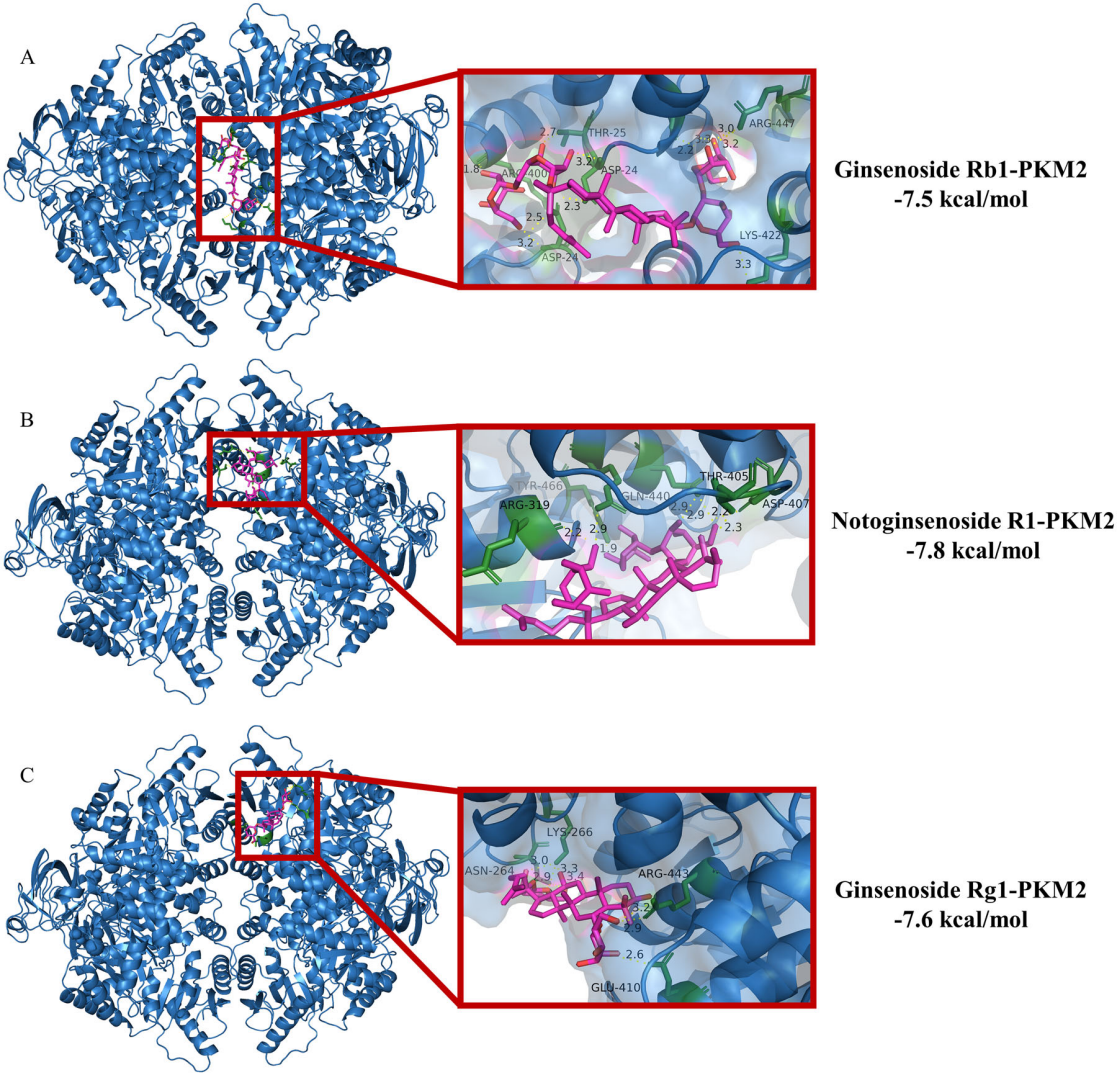
Interactions between PKM2 and ginsenoside Rb1, Rg1, and notoginsenoside R1 were examined by molecular docking. The interface is represented with a solid surface. PKM2 was represented with a cartoon and was colored blue. The binding residues was represented with cartoon and stick models and was colored green. Ginsenoside Rb1, Rg1, and notoginsenoside R1 was represented with sticks and was colored red. The hydrogen bonds were shown as yellow dotted lines.

Given the essential role of PKM2-mediated glycolysis in the differentiation of Th17 cells, we used molecular docking analysis to explore the potential binding mode between PNS and PKM2. PNS are active extracts obtained from the *Panax notoginseng*. Ginsenoside Rb1, ginsenoside Rg1, and notoginsenoside R1 are the main contributors of biological activities. Hence, we used these three saponins to molecular docking. Figure 3 shows that ginsenoside Rb1 binds to gaps in chains A, B, C and D of PKM2, and ginsenoside Rg1, notoginsenoside R1 binds to gaps in chains A and B of PKM2. The detailed analysis revealed that 11 hydrogen bond interactions were shown between ginsenoside Rb1 and residues ARG-447 (bond length: 2.2, 3.0, 3.2, 3.3 Å) and LYS-422 (bond length: 3.3 Å) and THR-25 (bond length: 2.7 Å) and ASP-24 (bond length: 2.3, 2.5, 3.2, 3.2 Å) and ARG-400 (bond length: 1.8 Å) of PKM2 (Figure 3(A)). Additionally, seven hydrogen bond interactions were also displayed between notoginsenoside R1 and residues THR-405 (bond length: 2.9, 2.9 Å) and ASP-407 (bond length: 2.2, 2.3 Å) and GLN-440 (bond length: 2.9 Å) and TYR-466 (bond length: 1.9 Å) and ARG-319 (bond length: 2.2 Å) of PKM2 (Figure 3(B)). Moreover, ginsenoside Rg1 formed 7 hydrogen bond interactions with residues LYS-266 (bond length: 2.9, 3.0 Å) and ASN-264 (bond length: 3.3, 3.4 Å) and ARG-443 (bond length: 2.9, 3.2 Å) and GLU-410 (bond length: 2.6 Å) of PKM2 (Figure 3(C)). These results described the main binding affinity between PNS and PKM2. Therefore, the molecular docking information illustrated that PKM2 could be a potential druggable target of PNS.

**Figure 4.**
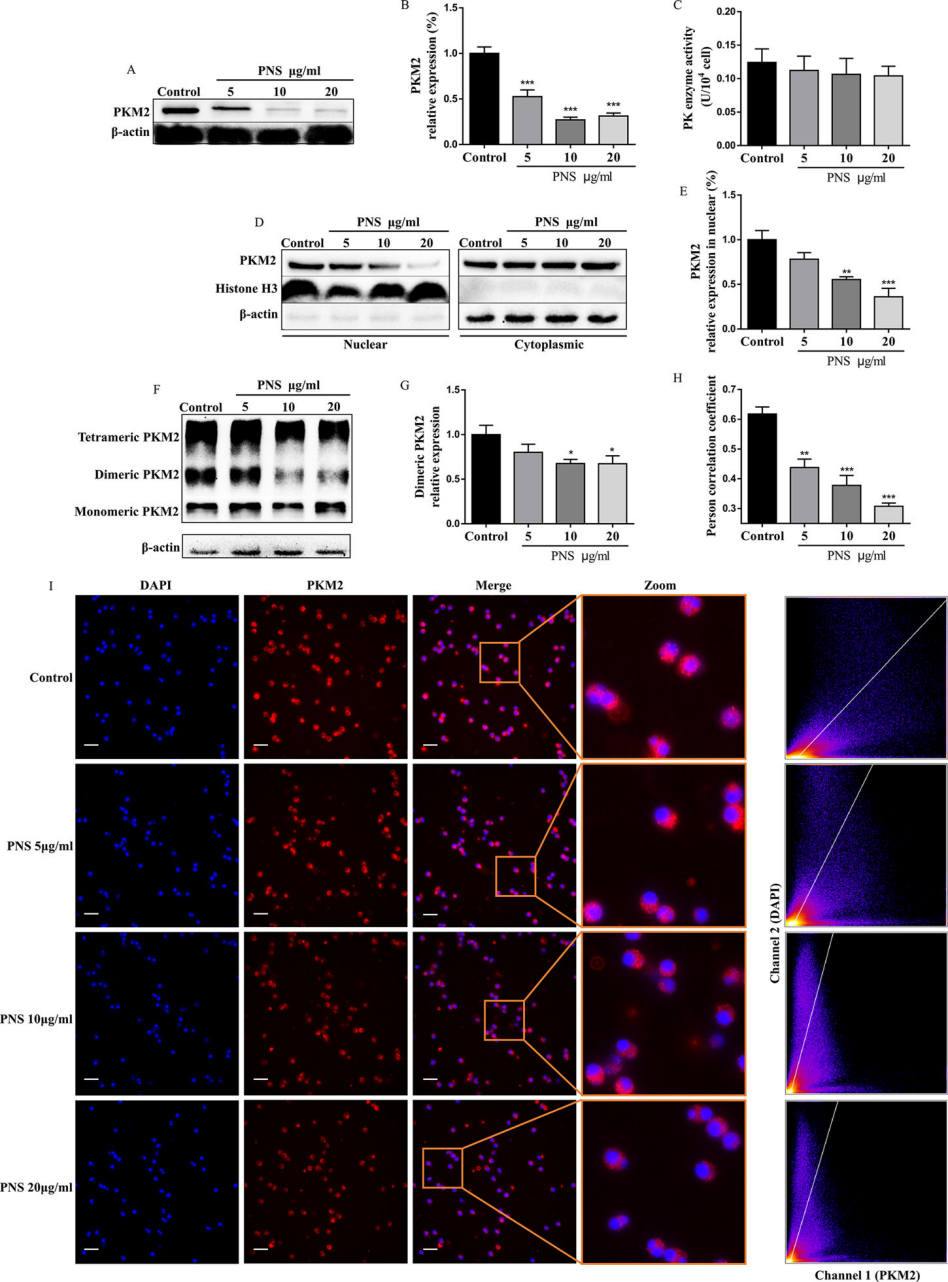
PNS inhibited PKM2 dimerization and blocked its nuclear accumulation. (A,B) The protein levels of PKM2 in CD4^+^T cells (with the condition of Th17-polarization) treated with PNS (5, 10 and 20 μg/mL) were evaluated by western blots. (C,D) CD4^+^T cells (with the condition of Th17-polarization) treated with PNS (5, 10 and 20 μg/mL), were collected and crosslinked with DSS, and analyzed for dimeric/tetrameric PKM2 expression by western blots. β-Actin was used as a loading control. (E,F) Nuclear and cytoplasmic fractions were isolated by cell fractionation from CD4^+^T cells (with the condition of Th17-polarization) treated with PNS (5, 10 and 20 μg/mL) and analyzed for PKM2 expression by western blots. Histone H3 and β-actin were used as nuclear and cytoplasm loading controls, respectively. (G,H) CD4^+^T cells (with the condition of Th17-polarization) were treated with or without PNS (5, 10 and 20 μg/mL). Subcellular localization of PKM2 were shown in left panel (scale bars, 10 μm). Cells were immunostained with anti-PKM2 (PKM2, red). The nucleus was marked with DAPI (blue). Merged images (Merge) were shown. The framed regions were zoomed in the fourth row (Zoom). The scatter diagram of PKM2 and DAPI signals were measured by Fiji ImageJ software. The co-localization of PKM2 and nucleus was expressed as person correlation coefficient determined by Fiji ImageJ software. Data were expressed as the mean ± SEM (*n* = 3). *p < 0.05, **p < 0.01, ***p < 0.001 compared with the control group by one-way ANOVA with Tukey’s *post hoc* test.

We next investigated whether PNS regulates PKM2 expression in Th17 cells. Strikingly, we observed that PNS (5, 10, 20 μg/mL) strongly decreased the protein expression of PKM2 (Figure 4(A,B)), while did not affect the metabolic enzyme activity of PK (Figure 4(C)). It has been reported that PKM2 is present in cells as tetramer or dimer. Dimeric PKM2 is enzymatically less active than the tetrameric isoform, which could translocate into the nucleus to favor the expression of genes associated with glycolysis (Israelsen and Vander Heiden 2015; Dong et al. 2016; Ma et al. 2019). We thus analyzed the effect of PNS on PKM2 conformation of Th17 cells. We first found that PNS significantly reduced the PKM2 dimeric isoform (Figure 4(F,G)), without a change in the tetrameric form of PKM2 upon Th17 cell differentiation (Figure S1A). In particular, the immunofluorescence imaging results suggested that PNS obviously prevented the co-localization of PKM2 and nucleus (Figure 4(H,I)). In accordance, PNS decreased the nuclear levels of PKM2 in Th17 cells, in a dose-dependent manner (Figures 4(D,E) and S1B). These results suggested that PNS inhibited PKM2 dimerization and blocked its nuclear accumulation.

### High PKM2 expression and nuclear accumulation upon Th17 cell differentiation

**Figure 5.**
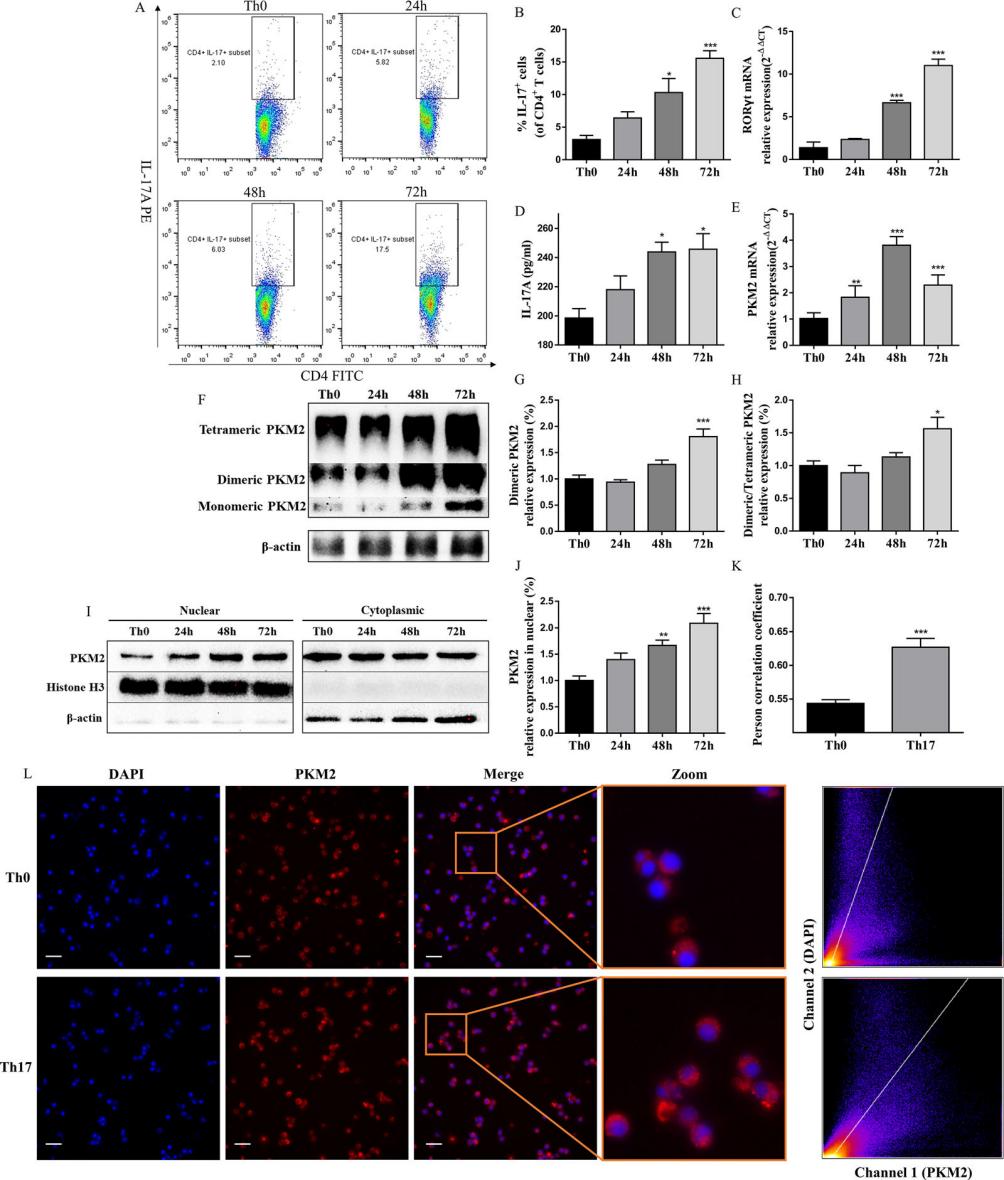
Th17 cell differentiation accompanied high PKM2 expression and nuclear accumulation. (A,B) Representative dot plots and the percentage of Th17 cells (CD4^+^IL-17^+^ cells) (with or without the condition of Th17-polarization) were determined by flow cytometry at different time points after culture. (C and E) The mRNA expression of RORγt and PKM2 were evaluated by RT-qPCR in CD4^+^T cells (with or without the condition of Th17-polarization) at different time points after culture. (D) The levels of IL-17A in Th17 cells at different time points after culture were measured by ELISA. (F–H) CD4^+^T cells (with or without the condition of Th17-polarization) were collected at different time points of differentiation, crosslinked with DSS, and analyzed for dimeric/tetrameric PKM2 expression by western blots. β-Actin was used as a loading control. (I,J) CD4^+^T cells (with or without the condition of Th17-polarization) were collected at different time points of differentiation. Nuclear and cytoplasmic fractions were isolated by cell fractionation and analyzed for PKM2 expression by western blots. Histone H3 and β-actin were used as nuclear and cytoplasm loading controls, respectively. (K,L) Subcellular localization of PKM2 were shown in left panel (scale bars, 10 μm). Cells were immunostained with anti-PKM2 (PKM2, red). The nucleus was marked with DAPI (blue). Merged images (Merge) were shown. The framed regions were zoomed in the fourth row (Zoom). The scatter diagram of PKM2 and DAPI signals were measured by Fiji ImageJ software. The co-localization of PKM2 and nucleus was expressed as person correlation coefficient determined by Fiji ImageJ software. Data were expressed as the mean ± SEM (*n* = 3). *p < 0.05, **p < 0.01, ***p < 0.001 compared with the control group by one-way ANOVA with Tukey’s *post hoc* test.

To determinate the role of PKM2 in the differentiation of Th17 cells, we analyzed PKM2 expression in CD4^+^T cells with or without the condition of Th17-polarization. The successful polarization, which significantly upregulated the percentage of CD4^+^IL-17^+^ cells, the RORγt mRNA expression, and the IL-17A levels, was verified by flow cytometry, qPCR, and ELISA (Figure 5(A–D)). As previously reported (Angiari et al. 2020), following Th17 cell differentiation, we observed an upregulation of PKM2 mRNA expression. Pkm2 mRNA expression was detectable at 24 h, and it reached a peak at 48 h of culture (Figure 5(E)). Interestingly, both the tetrameric and dimeric forms of PKM2 were also upregulated during the differentiation (Figures 5(F,G) and S2), and the expression of dimeric PKM2 were much higher throughout Th17 cell differentiation compared to tetrameric PKM2 (Figure 5(H)). We then examined subcellular localization of PKM2 upon Th17 cell differentiation. Immunofluorescent analysis showed that more PKM2 was translocated into nucleus during Th17 cell differentiation compared with Th0 cells (without the condition of Th17-polarization) (Figure 5(K,L)), which was support by western blots of nuclear fractionation, showing that the condition of Th17-polarization led to the time-dependent accumulation of PKM2 in the nucleus (Figure 5(I,J)). These data indicated a potential role for PKM2 non-metabolic functions in Th17 cell differentiation.

### PKM2 nuclear accumulation promoted Th17 cell differentiation and STAT3 phosphorylation

**Figure 6.**
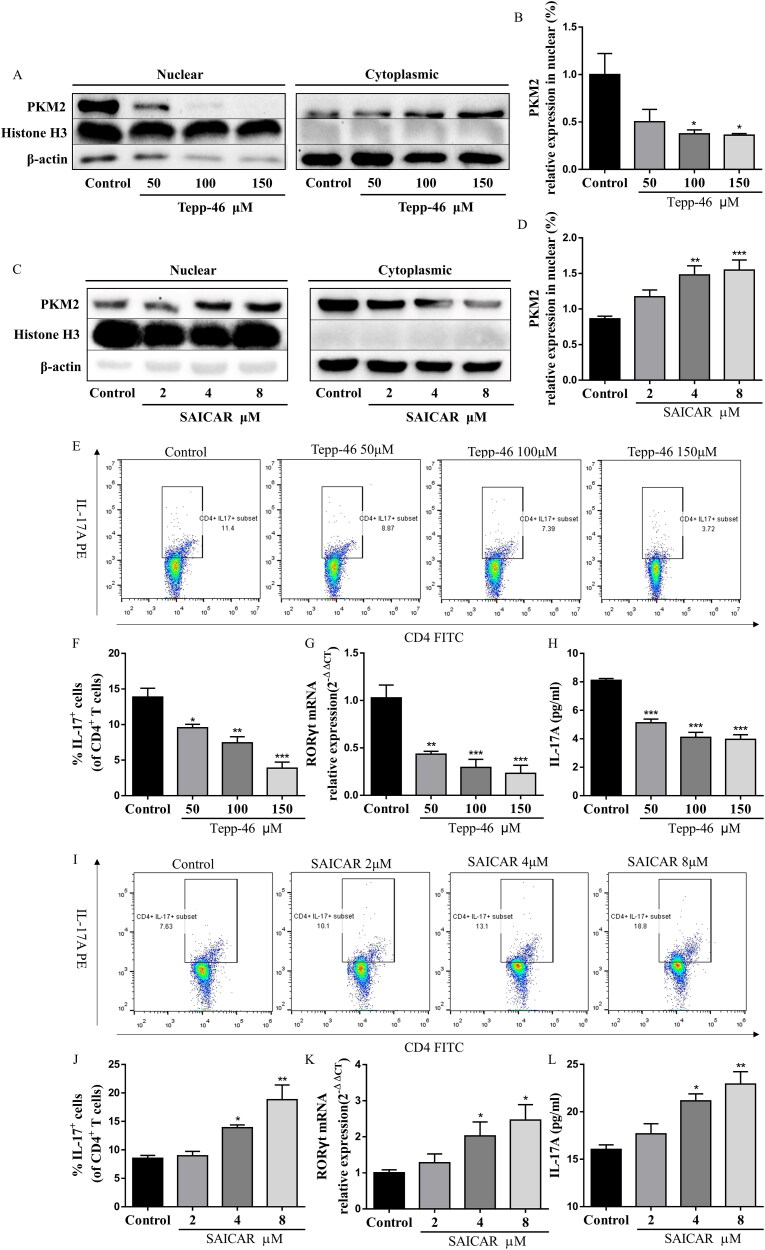
PKM2 nuclear accumulation promoted Th17 cell differentiation *in vitro*. (A,B) Representative dot plots and the percentage of Th17 cells (CD4^+^IL-17^+^ cells) (with the condition of Th17-polarization) treated with Tepp-46 (50, 100 and 150 μM) were determined by flow cytometry. (C) The mRNA expression of RORγt in CD4^+^T cells (with the condition of Th17-polarization) treated with Tepp-46 (50, 100 and 150 μM) was evaluated by RT-qPCR. (D) The IL-17A levels in medium of CD4^+^T cells (with the condition of Th17-polarization) treated with Tepp-46 (50, 100 and 150 μM) was detected by ELISA. (E,F) Representative dot plots and the percentage of Th17 cells (CD4^+^IL-17^+^ cells) (with the condition of Th17-polarization) treated with SAICAR (2, 4 and 8 μM) were determined by flow cytometry. (G) The mRNA expression of RORγt in CD4^+^T cells (with the condition of Th17-polarization) treated with SAICAR (2, 4 and 8 μM) was evaluated by RT-qPCR. (H) The IL-17A levels in medium of CD4^+^T cells (with the condition of Th17-polarization) treated with SAICAR (2, 4 and 8 μM) was detected by ELISA. Data were expressed as the mean ± SEM (*n* = 3). *p < 0.05, **p < 0.01, ***p < 0.001 compared with the control group by one-way ANOVA with Tukey’s *post hoc* test.

To investigate the functional importance of the PKM2 nuclear accumulation in mediating Th17 cell differentiation, we used the small molecule Tepp-46, which is a well-characterized PKM2-specific allosteric activator that promotes tetramer formation and inhibits nuclear accumulation (Anastasiou et al. 2012). Immunoblot analysis of nuclear fractions of Th17 cells showed that TEPP-46 reduced the nuclear levels of PKM2 in Th17 cells (Figure 6(A,B)), in a dose-dependent manner and had no cytotoxic effect at the three concentrations used (Figure S3A). Notably, treating CD4^+^T cells with TEPP-46 significantly reduced Th17 cell differentiation, including the percentage of CD4^+^IL-17^+^ cells and the RORγt mRNA expression and IL-17A levels (Figure 6(E–H)), suggesting that dimeric PKM2 nuclear accumulation was required for the regulation of Th17 cell differentiation. In addition, we also observed increased Th17 cell differentiation, RORγt mRNA expression and IL-17A levels upon treatment with SAICAR (Figure 6(I–L) and S3B), another well-characterized PKM2 allosteric inhibitor (Keller et al. 2012), which could cause its dimerization and promote its translocation into the nucleus (Figure 6(C,D)).

**Figure 7.**
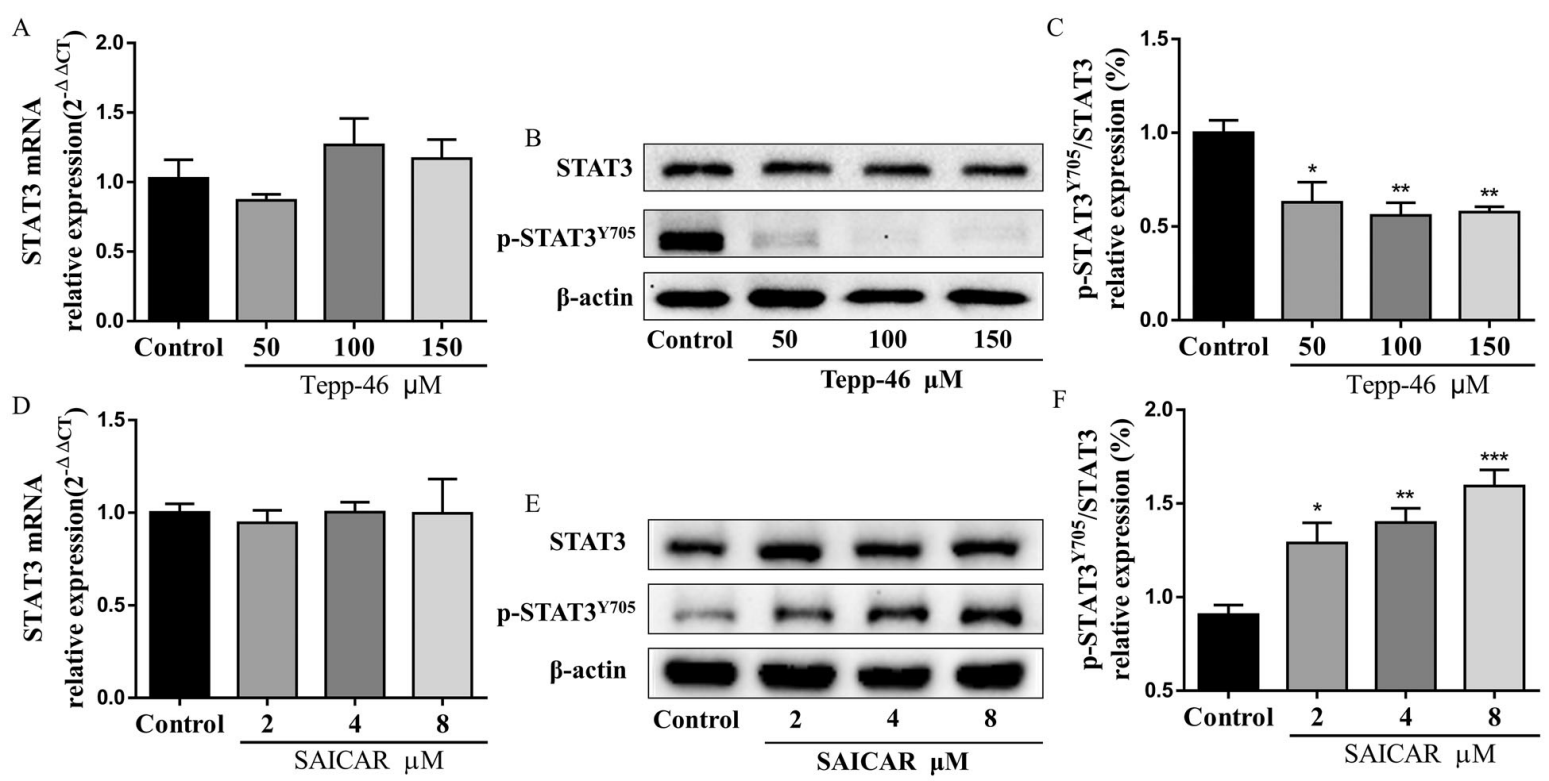
PKM2 nuclear accumulation promoted phospho-STAT3 at Y705 in Th17 cells. (A) The mRNA expression of STAT3 in CD4^+^T cells (with the condition of Th17-polarization) treated with Tepp-46 (50, 100, and 150 μM) was evaluated by RT-qPCR. (B,C) Tepp-46 (50, 100 and 150 μM)-treated Th17 cell lysates were subjected to western blot of total and phospho-STAT3 (Y705) expression. β-Actin was used as a loading control. (D) The mRNA expression of STAT3 in CD4^+^T cells (with the condition of Th17-polarization) treated with SAICAR (2, 4 and 8 μM) was evaluated by RT-qPCR. (E,F) SAICAR (2, 4 and 8 μM)-treated Th17 cell lysates were subjected to western blot of total and phospho-STAT3 (Y705) expression. β-Actin was used as a loading control. Data were expressed as the mean ± SEM (*n* = 3). *p < 0.05, **p < 0.01, ***p < 0.001 compared with the control group by one-way ANOVA with Tukey’s *post hoc* test.

Moreover, the phosphorylation of STAT3 (phospho-STAT3) at Y705 residue is known to be required for Th17 cell differentiation (Korn et al. 2009). Importantly, the nuclear dimeric PKM2 form can act as a protein kinase and phosphorylate STAT3 at Y705 in the nucleus of cancer cells and monocytes (Ma et al. 2019; Hou et al. 2020). Therefore, we focused on this pathway, and we confirmed the inhibition in Tepp-46-treated cells. Immunoblot analysis demonstrated that Tepp-46 substantially reduced the levels of Y705-phosphorylated STAT3 in Th17 cells (Figure 7(B,C)), while the total STAT3 protein expression and mRNA expression was not altered (Figure 7(A,B)). In accordance, SAICAR-treated Th17 cells showed significantly higher levels of phosphorylated STAT3 at Y705 (Figure 7(E,F)). Overall, these results indicated that dimeric PKM2 nuclear accumulation promoted phospho-STAT3 at Y705, a crucial step for Th17 cell differentiation.

### PNS inhibited Th17 cell differentiation in vitro via nuclear PKM2-mediated STAT3 phosphorylation

**Figure 8.**
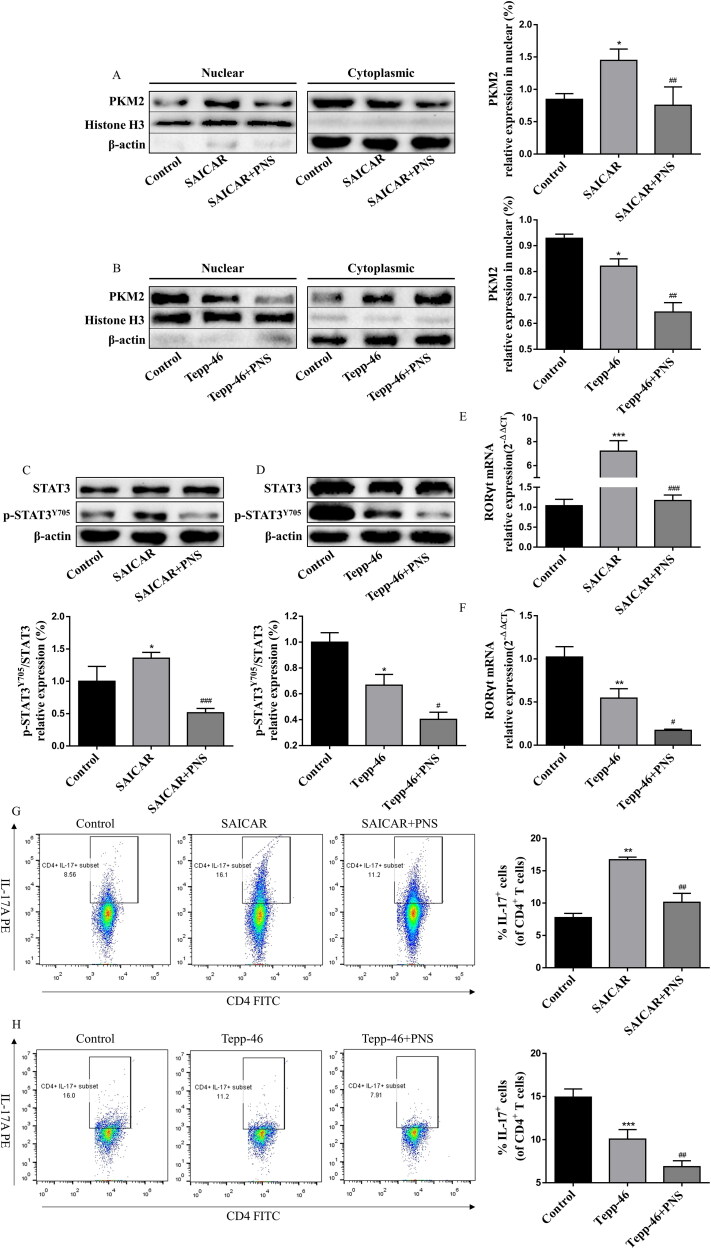
PNS blocked STAT3 phosphorylation and inhibited Th17 cell differentiation by suppressing PKM2 nuclear accumulation. Naïve CD4^+^T cells (with the condition of Th17-polarization) were pretreated with SAICAR (4 μM) or Tepp-46 (100 μM) for 24 h, and treated with PNS (10 μg/mL) for another 72 h. (A,B) Nuclear and cytoplasmic fractions were isolated by cell fractionation and analyzed for PKM2 expression by western blots. Histone H3 and β-actin were used as nuclear and cytoplasm loading controls, respectively. (C,D) The Th17 cell lysates were subjected to western blot of total and phospho-STAT3 (Y705) expression. β-Actin was used as a loading control. (E,F) The mRNA relative expression of RORγt was detected by RT-qPCR. (G,H) Representative dot plots and the percentage of Th17 cells (CD4^+^IL-17^+^ cells) were determined by flow cytometry. Data were expressed as the mean ± SEM (*n* = 3). *p < 0.05 compared with the control group, ^#^*p* < 0.05, ^##^*p* < 0.01 compared with the SAICAR or Tepp-46 group by one-way ANOVA with Tukey’s *post hoc* test.

As PNS treatment actually inhibits PKM2 nuclear accumulation and Th17 cell differentiation, we further investigated the underlying mechanism. We pretreated Th17 cells with SAICAR (4 μM) to induce PKM2 nuclear accumulation before PNS (10 μg/mL) treatment. As shown in Figure 8(A), SAICAR treatment successfully induced PKM2 nuclear accumulation in Th17 cells. We observed that PNS led to reduced levels of PKM2 nuclear accumulation (Figure 8(A)) and Y705-phosphorylated STAT3 in SAICAR-treated Th17 cells (Figure 8(C)). In addition, compared with Tepp-46 treatment (100 μM) alone, co-treatment with Tepp-46 and PNS significantly decreased PKM2 nuclear accumulation (Figure 8(B)) and Y705-phosphorylated STAT3 levels (Figure 8(D)), which indicated that the inhibition of PKM2 nuclear accumulation by PNS contributed to the disruption of STAT3 phosphorylation at Y705 in Th17 cells.

Afterwards, we determined whether the suppression of PKM2-mediated STAT3 phosphorylation by PNS inhibited Th17 cell differentiation. The flow cytometry and RT-qPCR results displayed that PNS reduced the RORγt mRNA expression (Figure 8(E)) and the percentage of CD4^+^IL-17^+^ cells (Figure 8(G)) after SAICAR pretreatment. In accordance, significantly decreased RORγt mRNA (Figure 8(F)) expression and CD4^+^IL-17^+^ cells percentage (Figure 8(H)) were observed after co-treatment with Tepp-46 and PNS, compared with Tepp-46 treatment alone. Collectively, our results suggested that PNS inhibited Th17 cell differentiation by blocking nuclear PKM2-mediated STAT3 phosphorylation.

### PNS repressed the PKM2/STAT3 signaling pathway to attenuate inflammation in CIA model

**Figure 9.**
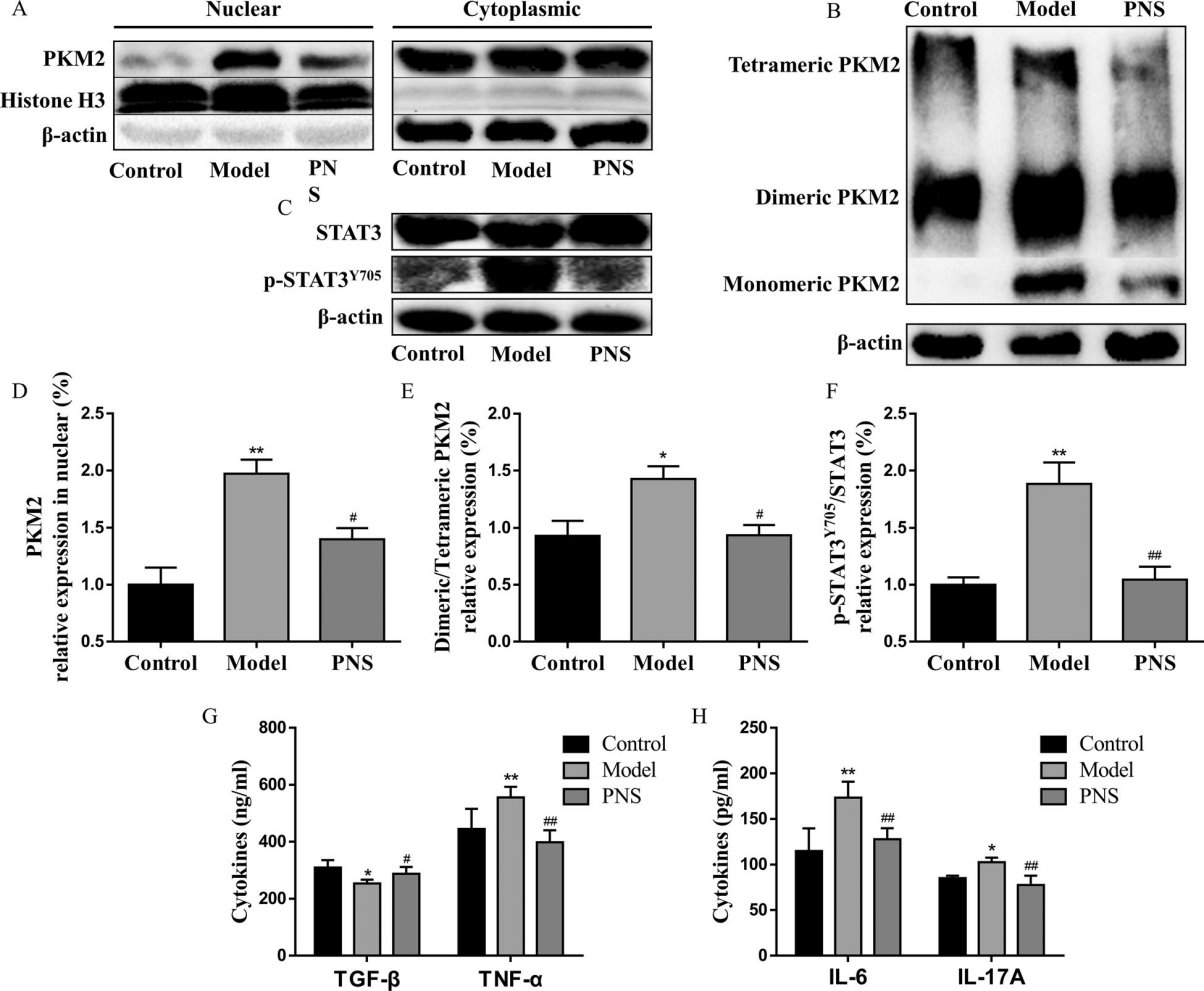
PNS repressed the PKM2/STAT3 signaling pathway to attenuate inflammation in the CIA model. DBA/1J mice were immunized with bovine type II collagen emulsified in complete Freund adjuvant and incomplete Freund adjuvant. Mice were treated at day 21 after the first immunization with saline or PNS (100 mg/kg). (A) Nuclear and cytoplasmic fractions were isolated by cell fractionation from splenic CD4^+^T cells of CIA mice and analyzed for PKM2 expression by western blots. Histone H3 and β-actin were used as nuclear and cytoplasm loading controls, respectively. (B) Splenic CD4^+^T cells from CIA mice were crosslinked with DSS and analyzed for dimeric/tetrameric PKM2 expression by western blots. β-Actin was used as a loading control. (C) Splenic CD4^+^T cell lysates from CIA mice were subjected to a western blot of total and phospho-STAT3 (Y705) expression. β-Actin was used as a loading control. Data were expressed as the mean ± SEM (*n* = 6). *p < 0.05, **p < 0.01, compared with the control group, ^#^*p* < 0.05, ^##^*p* < 0.01, compared with the model group, by one-way ANOVA with Tukey’s *post hoc* test.

Given the ability of Th17 cell differentiation to induce inflammation in CIA mice, we further investigated the effects of PNS on PKM2/STAT3-induced inflammation in CIA mice. The expression of dimeric PKM2 and its nuclear accumulation was upregulated in splenic CD4^+^T cells of CIA mice (Figure 9(A,B)), followed by phosphorylation of STAT3 at Y705 (Figure 9(C)). PNS treatment exerted significant inhibitory effects on PKM2/STAT3 signaling pathway (Figure 9(A–F)). Subsequently, we observed marked reductions in TNF-α, IL-6, and IL-17A (pro-inflammatory cytokines) and an increase in TGF-β (anti-inflammatory cytokines) in the serum compared with those of the model group (Figure 9(G,H)). Our results indicated that PNS blocking PKM2 dimerization and nuclear accumulation could be considered as a potential therapeutic approach for the treatment of Th17-mediated pathologies.

## Discussion

Rheumatoid arthritis (RA) is an autoimmune disease characterized by synovial inflammation and the formation of pannus. The development and pathogenesis of RA is attributed to the association of several types of cells, including T cells, B cells, and autoantibodies (Ahmad et al. 2017).

Th17 cells are one of the key effectors of most common autoimmune diseases, including RA and multiple sclerosis (MS) (Korn et al. 2009; van Hamburg and Tas 2018). Th17 cells largely influence the development and progression both in human subjects and mice models of EVE through the release of signature cytokines such as IL-17A/IL-22 (Alhazzani et al. 2021). In asthmatic patients, IL-17 expression is increased in the lungs, sputum, bronchoalveolar lavage (BAL) fluids, or sera, and the severity of airway hypersensitivity in patients correlates with IL-17 expression level. Th17-derived cytokines, such as IL-17A not only regulate neutrophilic inflammation but also enhances Th2 airway responses (Nadeem et al.^2018^). However, the plasticity of Th17 cell differentiation and the synergistic effect of multiple inflammatory cytokines which produced during differentiation, such as IL-17, TNF, and interferon-γ (IFN-γ), are very important to RA pathogenesis (Buck et al. 2015). These might be the reasons why anti-IL-17 antibodies, such as secukinumab or ixekizumab, slightly affect the disease activity of RA (Fragoulis et al. 2016; Blanco et al. 2017). Thus, manipulating the Th17 cell pathway, as opposed to a single effector cytokine, will show superior efficacy in RA. PNS are the main bioactive compounds in *Panax notoginseng*, accounting for 8-12% of its total dry weight (Wang et al. 2016). A considerable clinical effect in the relief of RA inflammation and joint pain by PNS has been described (Li 1991; Li et al. 2010), but the regulatory mechanisms have not been elucidated. Some studies showed that PNS-induced regulation of osteoclastogenesis is responsible, in part, for its bone-protective effects (Jang et al. 2011). And a previous study indicated that the therapeutical effect of PNS may be associated with a decrease in the number of Th17 cells and the inhibition in the production of IL-17 (Wei JR et al. 2017). In accordance, our data displayed that PNS obviously decreased arthritis score and paw thickness, and attenuated inflammation in CIA mice. Meanwhile, the differentiation of Th17 cells have also been down-regulated by PNS treatment *in vivo* and *vitro*. These data supported the therapeutic role of PNS in the treatment of Th17-mediated RA. However, deeply exploring the underlying mechanisms is urgent.

Cell metabolism is important in T cell differentiation and Th17 cells mainly use glycolysis, this is owing to that glycolysis produces energy and biomacromolecules quickly compared with that produced by the tricarboxylic acid cycle (Shen et al. 2017; Okano et al. 2018; Weyand and Goronzy 2020). Due to cell metabolism-regulating T cell fate, modulation of the glycolysis pathway has been considered as a potential novel therapeutic approach for Th17-mediated diseases. PKM2 is the final rate-limiting enzyme in glycolysis, catalyzing the conversion of PEP to pyruvate, and is upregulated in Th17 cells (Angiari et al. 2020). Previous studies have displayed that PKM2 is requisite for Th17 cell differentiation. Silencing of PKM2 reduced glycolysis and differentiation to Th17 cells, while PKM2 overexpression restored Th17 cell differentiation (Kono et al. 2019). Therefore, we evaluated the regulatory function on PKM2 of PNS. The molecular docking information indicated the main binding affinity between PKM2 and ginsenoside Rb1, Rg1, notoginsenoside R1. Surprisingly, PNS strongly decreased the protein expression of PKM2, while it did not affect PK activity, which revealed that PNS might not fully block the glycolysis pathway although it inhibited PKM2 expression. Of note, emerging evidence proposed that metabolic enzymes, rather than solely being components of biochemical pathways, are also proteins that mediate many other moonlighting activities, such as gene transcription and protein kinase activity (Prakasam et al. 2018). Tetrameric PKM2 exerts highly enzymatical activity in the cytoplasm, while dimeric PKM2 translocates to the nucleus of proliferating cells for exerting non-metabolic functions (Israelsen and Vander Heiden 2015; Wu et al. 2020). For these reasons, we speculated that changes of PKM2 conformation is likely to play a significant role in PNS regulating Th17 cell differentiation. However, further studies are required to shed light on this aspect.

In our study, we reported that dimeric PKM2 expression was higher in differentiated Th17 cells than tetrameric PKM2 form, with a concomitant upregulation of PKM2 expression in the nucleus, in agreement with data from a previous report (Angiari et al. 2020), revealing a potential role for PKM2 non-metabolic functions in Th17 cell differentiation. Besides, our work also suggested that inhibition of PKM2 dimerization and nuclear accumulation by Tepp-46 constrained the differentiation of Th17 cells while promoting dimeric PKM2 nuclear translocation by SAICAR upregulated the number of Th17 cells. These data demonstrated that dimeric PKM2 nuclear accumulation was required for the regulation of Th17 cell differentiation. Importantly, PNS significantly reduced the PKM2 dimeric isoform, without a change in the tetrameric form of PKM2, and obviously decreased the nuclear expression of PKM2 upon Th17 cell differentiation, indicating that the underlying molecular mechanism is associated with the inhibition of PKM2 nuclear accumulation. Co-treatment with PNS and Tepp-46/SAICAR further suggested that PNS inhibited Th17 cell differentiation by suppressing dimeric PKM2 nuclear accumulation. These results were supported by the inhibitory effect we observed in CIA mice.

## Conclusions

**Figure 10.**
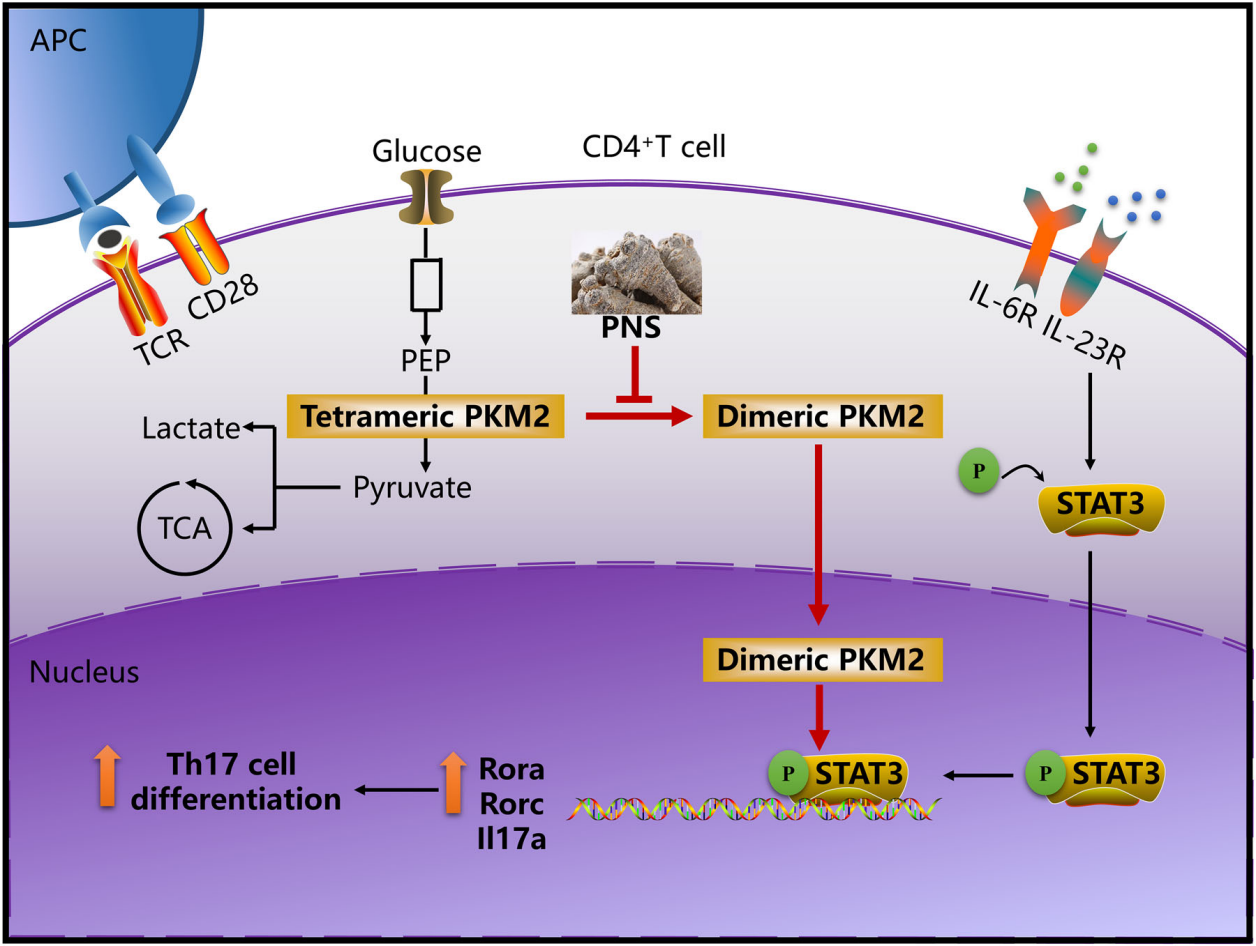
PNS attenuates Th17 cell differentiation *via* inhibition of nuclear PKM2-mediated STAT3 phosphorylation. The cooperation between TCR activation and costimulatory signals leads to a significant increase of PKM2 expression. IL-6 and IL-23 are the important cytokines for controlling the Th17 cell differentiation, IL-6R and IL-23R signaling cascade promote STAT3 phosphorylation/activation, companying with an accumulation of dimeric PKM2 in Th17 cells. The dimeric oligomer state facilitates PKM2 translocation into the nucleus and enhancing STAT3 phosphorylation, contributing to increase its transcriptional activity. This process ultimately enhances the transcription of Th17 cell–associated genes. PNS specifically inhibit PKM2 dimerization and nuclear accumulation, and further induced the decrease of STAT3 phosphorylation, contributing to suppress Th17 cell differentiation.

Dimeric PKM2 has been shown to translocate into the nucleus and enhance STAT3 phosphorylation at Y705, contributing to cancer cell proliferation and inflammatory cytokine production in macrophages (Ma et al. 2019; Hou et al. 2020). Moreover, IL-6 and TGF-β promote Th17 cell differentiation through activating the STAT3 signaling pathway (Chen et al. 2006; Korn et al. 2009). Thus, we demonstrated that inhibition of nuclear accumulation of dimeric PKM2 down-regulated STAT3 phosphorylation at Y705 in Th17 cells. In agreement with our findings, in the course of our study, a study was published supporting the role of nuclear PKM2-mediated STAT3 phosphorylation upon Th17 cell differentiation (Damasceno et al. 2020). In brief, our data suggested that dimeric PKM2 nuclear accumulation promoted phospho-STAT3 at Y705, a crucial step for Th17 cell differentiation. Furthermore, we observed that PNS led to reduced levels of Y705-phosphorylated STAT3 in SAICAR-treated Th17 cells, indicating that PNS inhibit Th17 cell differentiation by blocking nuclear PKM2-mediated STAT3 phosphorylation. The data from the *in vivo* study also confirmed that PNS alleviated Th17-mediated inflammatory responses by inhibiting the PKM2/STAT3 signal pathway (Figure 10).

Although glycolysis is essential for Th17 cell differentiation, it is a universal metabolic pathway whose blockade by inhibitors such as 2-deoxy-d-glucose (2DG) can yield adverse side effects (Raez et al. 2013). Thus, the drugs that fully inhibit the glycolysis pathway are not likely to be useful in clinical treatment. We report that PNS inhibited Th17 cell differentiation *via* blocking dimeric PKM2 translocating into the nucleus and STAT3 phosphorylation, without suppressing glycolytic activity and cell function. Indeed, specific inhibition of non-metabolic functions of PKM2 by PNS contributes to the therapeutic benefit of RA without impacting basal metabolic functions. In summary, this work demonstrated that PNS inhibited Th17 cell differentiation through the inhibition of nuclear PKM2-mediated STAT3 phosphorylation.

## Supplementary Material

Supplemental Material
