# Supplementary material for: Panax notoginseng saponins (PNS) attenuate Th17 cell differentiation in CIA mice via inhibition of nuclear PKM2-mediated STAT3 phosphorylation
Source: Pharm Biol. 2023 Feb 16;61(1):459–72. doi: 10.1080/13880209.2023.2173248 (PMC9936999; doi:10.1080/13880209.2023.2173248)
Supplement: Supplemental Material [file IPHB_A_2173248_SM2358.docx]

**SUPPLEMENTARY MATERIAL**

**PNS attenuate Th17 cell differentiation in rheumatoid arthritis via inhibition of nuclear PKM2-mediated STAT3 phosphorylation**

**Mei-Yu Shen^1^, Yu-Xi Di^1^, Xiang Wang^1^, Feng-Xiang Tian^1^, Ming-Fei Zhang^1^, Fei-Ya Qian^1^, Bao-Ping Jiang^1^, Xue-Ping Zhou^2,3^, Ling-Ling Zhou^1*^**

^1^School of Pharmacy, Jiangsu Key Laboratory for Pharmacology and Safety Evaluation of Chinese Materia Medica, Nanjing University of Chinese Medicine, NO.138 Xianlin Road, 210023, Nanjing, Jiangsu province, PR China.

^2^Department of Rheumatology, Affiliated Hospital of Nanjing University of Chinese Medicine, NO.155 Hanzhong Road, 210029, Nanjing, Jiangsu province, PR China.

^3^The First Clinical Medical College, Nanjing University of Chinese Medicine, NO.138 Xianlin Road, 210023, Nanjing, Jiangsu province, PR China.

* Corresponding author. Ling-Ling Zhou. (E-mail: zhoulingling@njucm.edu.cn)

**
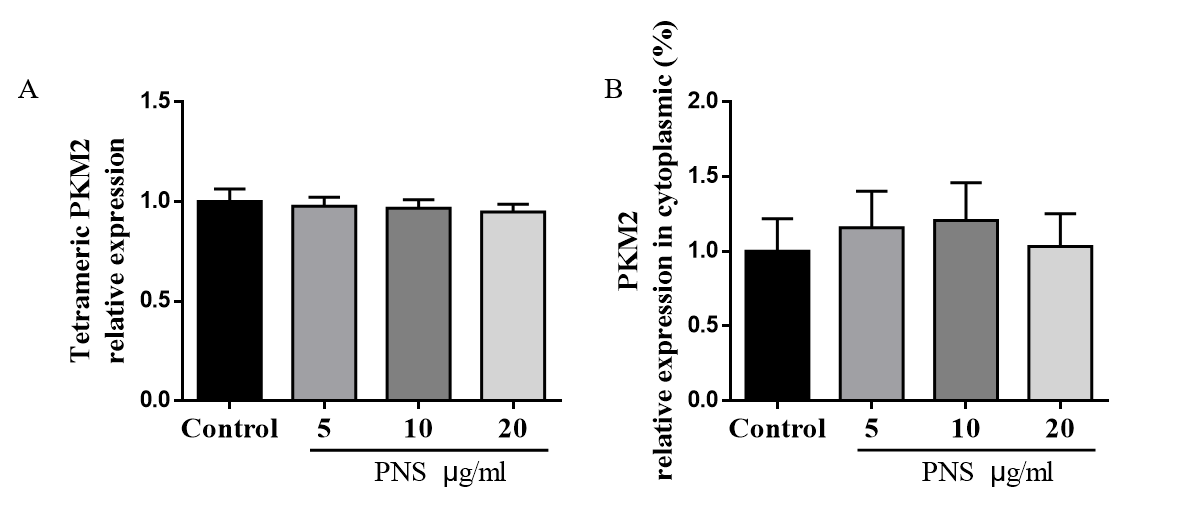
**

**Fig. S1: PNS did not affect the levels of tetrameric PKM2 in cytoplasmic.**

(A) CD4^+^T cells (with the condition of Th17-polarization) treated with PNS (5, 10 and 20 μg/ml), were collected and crosslinked with DSS, and analyzed for tetrameric PKM2 expression by western blots. (B) Cytoplasmic fractions were isolated by cell fractionation from CD4^+^T cells (with the condition of Th17-polarization) treated with PNS (5, 10 and 20 μg/ml) and analyzed for PKM2 expression by western blots.

Data were expressed as the mean ± SEM (n=3). *p < 0.05, **p < 0.01, ***p < 0.001 compared with the control group by one-way ANOVA with Tukey’s post-hoc test.


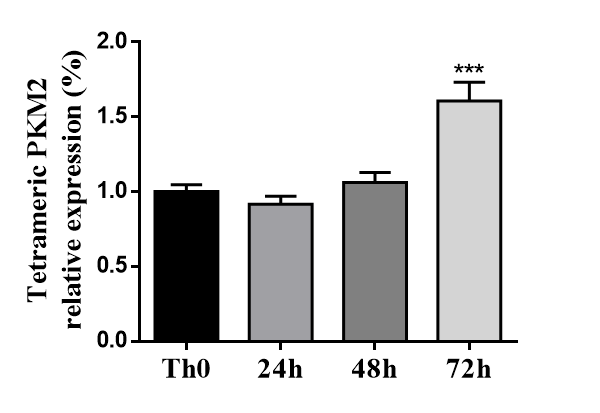


**Fig. S2: Th17 cell differentiation accompanied high tetrameric PKM2 expression.**

CD4^+^T cells (with or without the condition of Th17-polarization) were collected at different time points of differentiation, crosslinked with DSS, and analyzed for tetrameric PKM2 expression by western blots. Data were expressed as the mean ± SEM (n=3). *p < 0.05, **p < 0.01, ***p < 0.001 compared with the control group by one-way ANOVA with Tukey’s post-hoc test.


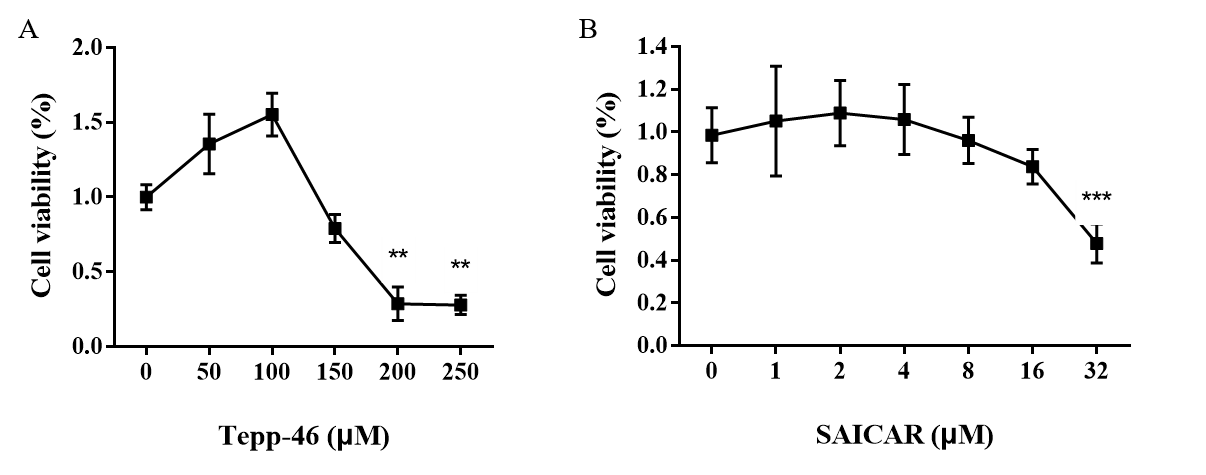


**Fig. S3: The cell viability of CD4^+^T cells (with the condition of Th17-polarization) treated with Tepp-46 or SAICAR was detected by CCK8.**

Data were expressed as the mean ± SEM (n=3). *p < 0.05, **p < 0.01, ***p < 0.001 compared with the control group by one-way ANOVA with Tukey’s post-hoc test.
